# Pharmacological Update Properties of *Aloe Vera* and its Major Active Constituents

**DOI:** 10.3390/molecules25061324

**Published:** 2020-03-13

**Authors:** Marta Sánchez, Elena González-Burgos, Irene Iglesias, M. Pilar Gómez-Serranillos

**Affiliations:** Department of Pharmacology, Pharmacognosy and Botany, Faculty of Pharmacy, Universidad Complutense de Madrid (UCM), 28040 Madrid, Spain; martas15@ucm.es (M.S.); elenagon@ucm.es (E.G.-B.); ireneig@ucm.es (I.I.)

**Keywords:** *Aloe vera*, pharmacology, extracts, isolated compounds

## Abstract

*Aloe vera* has been traditionally used to treat skin injuries (burns, cuts, insect bites, and eczemas) and digestive problems because its anti-inflammatory, antimicrobial, and wound healing properties. Research on this medicinal plant has been aimed at validating traditional uses and deepening the mechanism of action, identifying the compounds responsible for these activities. The most investigated active compounds are aloe-emodin, aloin, aloesin, emodin, and acemannan. Likewise, new actions have been investigated for *Aloe vera* and its active compounds. This review provides an overview of current pharmacological studies (in vitro, in vivo, and clinical trials), written in English during the last six years (2014–2019). In particular, new pharmacological data research has shown that most studies refer to anti-cancer action, skin and digestive protective activity, and antimicrobial properties. Most recent works are in vitro and in vivo. Clinical trials have been conducted just with *Aloe vera*, but not with isolated compounds; therefore, it would be interesting to study the clinical effect of relevant metabolites in different human conditions and pathologies. The promising results of these studies in basic research encourage a greater number of clinical trials to test the clinical application of *Aloe vera* and its main compounds, particularly on bone protection, cancer, and diabetes.

## 1. Introduction

*Aloe vera* (*Aloe barbadensis* Miller, family Xanthorrhoeaceae) is a perennial green herb with bright yellow tubular flowers that is extensively distributed in hot and dry areas of North Africa, the Middle East of Asia, the Southern Mediterranean, and the Canary Islands. *Aloe vera* derives from “Allaeh” (Arabic word that means “shining bitter substances”) and “Vera” (Latin word that means “true”). The colorless mucilaginous gel from *Aloe vera* leaves has been extensively used with pharmacological and cosmetic applications. Traditionally, this medicinal plant has been employed to treat skin problems (burns, wounds, and anti-inflammatory processes). Moreover, *Aloe vera* has shown other therapeutic properties including anticancer, antioxidant, antidiabetic, and antihyperlipidemic. *Aloe vera* contains more than 75 different compounds, including vitamins (vitamin A, C, E, and B12), enzymes (i.e., amylase, catalase, and peroxidase), minerals (i.e., zinc, copper, selenium, and calcium), sugars (monosaccharides such as mannose-6-phosphate and polysaccharides such as glucomannans), anthraquinones (aloin and emodin), fatty acids (i.e., lupeol and campesterol), hormones (auxins and gibberellins), and others (i.e., salicylic acid, lignin, and saponins) [1,2,3].

In this review, we summarize an update of the pharmacological activities (in vitro, in vivo, and clinical trials) of *Aloe vera*. Publications (original papers) were published in English in the years 2014 to 2019 in peer-reviewed scientific journals of the Pubmed database. Those articles that included *Aloe vera* combined with other plants or *Aloe* species other than *Aloe vera* were excluded from this review.

**Figure 1:**
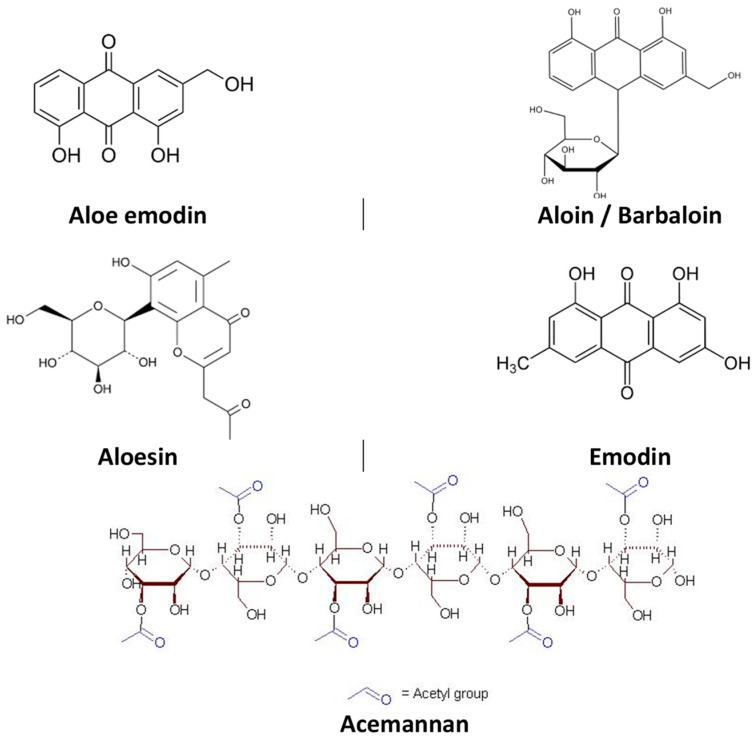
Chemical structure of compounds isolated from *Aloe vera* with pharmacological activity.

**Figure 2:**
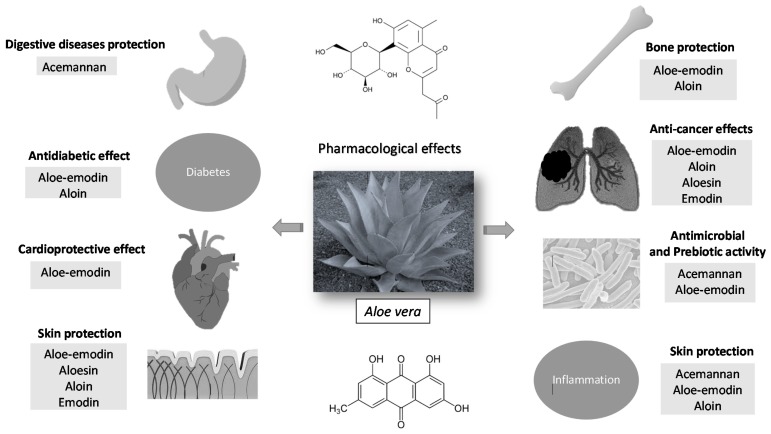
Pharmacological effects of the main constituents of *Aloe vera*.

This review is structured into different activities, which include in vitro, in vivo, and clinical trials, published in the last six years. The order of activities is based on the interest and importance of studies for *Aloe vera*. The Table 1 (in vitro studies), Table 2 (in vivo studies), and Table 3 (clinical trials) summarize the main pharmacological findings for *Aloe vera* and its isolated compounds (Figure 1, Figure 2).

## 2. Digestive Diseases Protection

*Aloe vera* extract (50%) increased cell viability of dental pulp stem cells being useful for avulsed broken teeth [4]. This effect is attributed to polysaccharides, mainly acemannan, by inducing osteogenic-specific gene expressions, DNA synthesis, growth factor, and JAK-STAT pathway [5,6]. Moreover, *Aloe vera* (225 mg/kg) exerted a radioprotective effect against salivary gland dysfunction in a rat model as evidenced in an increase of salivary flow rate [7].

Periodontitis is a serious and common dental affliction in which gums are infected and become inflamed, causing tissue and bone destruction. Gingivitis is the initial phase of periodontitis and is caused by dental plaque. Significant clinical evidence has demonstrated that *Aloe vera* mouthwash and gel are effective in the prevention and treatment of gingivitis and periodontitis by reducing gingival index, plaque index, and probing depth and by increasing bone fill and regeneration [8,9,10,11,12,13,14]. *Aloe vera* has proven to be as effective as other usual treatments such as chlorhexidine, alendronate, and chlorine dioxide [8,10,11,13].

In a randomized placebo double-blind study with 20 healthy adults, Fallahi et al. [15] investigated the effect of *Aloe vera* mouthwash on postoperative complications after impacted third molar surgery. *Aloe vera* gel significantly reduced swelling and postoperative pain. In another work, Kalra et al. [16] evaluated the efficacy of *Aloe vera* gel and mineral trioxide aggregate as pulpotomy agents in primary molar teeth. The overall success rates at 3, 6, 9, and 12 months was high for patients treated with mineral trioxide aggregate. Moreover, a cross-sectional randomized interventional study revealed that *Aloe vera* gel promoted wound healing and reduced pain in patients that required atraumatic tooth extractions, and its effectiveness was higher than that of traditional analgesics [17]. Furthermore, *Aloe vera* resulted to be a promising cavity disinfecting agent in minimally invasive dentistry in a randomized clinical trial with 10 patients [18].

Oral mucositis/stomatitis is an inflammatory and/or ulcerative condition that occurs as a debilitating complication of chemotherapy and radiotherapy treatments and affects quality of life of oncological patients. *Aloe vera* mouthwash alleviated radiation-induced mucositis severity in patients with head and neck cancers similarly to the reference benzydamine mouthwash [19]. Moreover, *Aloe vera* mouthwash has also demonstrated to be efficient in the treatment of stomatitis (mean intensity and pain) associated with radiotherapy in patients with acute myeloid leukemia and acute lymphocytic leukemia [20].

Oral submucous fibrosis is a precancerous condition of the oral cavity characterized by abnormal collagen deposition. This malignant disorder is mainly caused by chewing areca nut and it is most frequent in India and Southeast Asia. Anuradha et al. [21] evaluated the efficacy of *Aloe vera* (systemic as juice and topical as gel) in the treatment of oral submucous fibrosis. Clinical evidence demonstrated that *Aloe vera* reduced burning sensation and increased cheek flexibility, mouth opening, and tongue protrusion similar to the reference treatment hydrocortisone, hyaluronidase, and antioxidant supplements. In another study on oral submucous fibrosis, the combination of *Aloe vera* gel with physiotherapy was more efficient in decreasing burning sensation and increasing tongue protrusion, mouth opening, and cheek flexibility than the combination of antioxidant capsules with physiotherapy [22].

Gastroesophageal reflux disease is a common chronic digestive disease in which gastric acids move up into the esophagus. *Aloe vera* syrup (10 mL/day) for 4 weeks reduced the frequency of symptoms of gastroesophageal reflux diseases including heartburn, food regurgitation, dysphagia, flatulence, belching, nausea, and acid regurgitation without causing adverse effects (only one case of vertigo and another of stomach ache were reported) [23].

Gastritis is an inflammation of mucous membrane layer of the stomach. *Aloe vera* gel protected in a Balb/c mouse model of alcohol-induced acute gastritis by increasing matrix metalloproteinase-9 inhibitory activity [24].

The topical administration of *Aloe vera* 3% ointment alleviated the symptoms of diarrhea and fecal urgency in patients with acute radiation proctitis induced by radiotherapy of the pelvic area [25]. Moreover, *Aloe barbadensis* extract (AVH200^®^) reduced, but not significantly, the severity of gastrointestinal symptoms in patients with irritable bowel syndrome compared to a control group [26]. Lin et al. [5] revealed that *Aloe* polysaccharide (15 mg/kg) protected rats from 2,4,6-three nitrobenzene sulfonic acid colitis induced by increasing JAK2, p-JAK2, STAT-3, and p-STAT3 protein expression. Furthermore, *Aloe vera* cream applied three times daily for 6 weeks reduced chronic anal fissure pain and hemorrhaging after defection and promoted wound healing in a prospective double blind clinical trial [27].

## 3. Skin Protection

Most in vitro studies on skin protection study the ability of *Aloe vera* and active compounds in wound healing. The immortalized human keratinocyte HaCaT cell line, the primary normal human epidermal keratinocytes HEKa cell line, and fibroblasts cell lines are the most used. These studies have revealed that *Aloe vera* and its major compounds (aloesin, aloin, and emodin) exert their protective action mainly through antioxidant and anti-inflammatory mechanisms. Hence, *Aloe vera* up-regulated TFGβ1, bFGF, and Vegf-A expression in fibroblasts and increased keratinocyte proliferation and differentiation by lysosomal membrane stability [28,29,30,31,32]. Moreover, *Aloe vera* solution could accelerate corneal wound closure at low concentrations (≤175 μg/mL) by increasing type IV collagen-degrading activity in a cellular model of primary cultures of corneal epithelial cells [33]. Furthermore, aloin exerted skin protection by reducing IL-8 production, DNA damage, lipid peroxidation, and ROS generation and by increasing GSH content and SOD activity [34]. The compound aloesin resulted in promoting wound healing by increasing cell migration via phosphorylation of Cdc42 and Rak1, cytokines, and growth factors [35]. In addition to this healing activity, it has been seen that *Aloe* polysaccharide (20, 40, and 80 µg/mL for 24 h) could be a beneficial agent in psoriasis as evidenced in the inhibition of TNF-α levels and IL-8 and IL-12 protein expression in human keratinocyte HaCaT cell line.

As for in vivo studies, the most common models are genetically modified animals (BALB/c mice, HR-1 hairless mice and SKH-1 hairless mice) and UV and X-ray skin damage in animals. Most of these in vivo studies have been done with *Aloe vera* extracts and gel. Application of topical *Aloe vera* favored wound healing in animal models with dermal incisions by reducing inflammatory cell infiltration, increasing CD4+/CD8+ ratio lymphocytes, and improving epidermal thickness and collagen deposition [36,37,38,39]. In another study conducted in Indonesia with several medicinal plants, the effect of *Nigella sativa* oil gel and *Aloe vera* gel to treat diabetic ulcers was investigated. *Aloe vera* resulted to be more efficient in improving wound healing on alloxan-induced diabetes in Wistar rats with wounds on dorsum as evidenced by a decrease of necrotic tissue and inflammation and an improvement of re-epithelialization [40]. Furthermore, a UV-induced mice model revealed that *Aloe vera* gel powder increased epidermal growth factor and hyaluronan synthase and reduced matrix metalloproteinases expression (types 2, 9, and 13) [41,42]. *Aloe* sterols are involved in this UV protection [43]. Likewise, it has been observed that *Aloe vera* protected against X-radiation through antioxidant mechanisms (increased antioxidant enzyme activity and GSH content and reduced ROS production and lipid peroxidation) [44,45]. Among isolated compounds, investigations with the compounds aloe-emodin and aloesin have shown that their healing activity is due to angiogenic properties [46,47].

In the last 6 years, several clinical trials have also been carried out. Some of these have been aimed at evaluating the effectiveness of *Aloe vera* on ulcers. Hence, the administration of *Aloe vera* gel twice daily for 3 months improved and accelerated wound healing as well as reduced hospitalization time [48,49]. Moreover, in a randomized, triple-blind clinical trial with 80 patients hospitalized in the orthopedic ward, Hekmatpou et al. [50] demonstrated that *Aloe vera* gel twice daily for 10 days prevented the development of pressure ulcers on the areas of hip, sacrum, and heel. Moreover, clinical trials have demonstrated that *Aloe vera* facilitated rapid tissue epithelialization and granulation in burns [51], promoted healing of cesarean wound [52], and accelerated wound healing of split-thickness skin graft donor sites [53]. Furthermore, *Aloe vera* has been investigated in randomized, double-blind, placebo-controlled studies for its benefits to maintain healthy skin. Therefore, the daily oral intake of 40 µg of *Aloe* sterol (cycloartenol and lophenol) for at least 12 weeks improved skin elasticity in men under 46 years exposed to the sunlight but do not use sunscreen to protect themselves [54], reduced facial wrinkles in Japanese women over 40 years old by stimulating hyaluronic acid and collagen production [55], and increased gross elasticity, net elasticity, and biological elasticity in women aged 30–59 [56]. However, despite clinical evidence on the protective role of *Aloe vera* in the skin, there are clinical trials that have not yet found effectiveness of this medicinal plant, particularly in decreasing radiation-induced skin injury. Two clinical trials have been published between 2014 and 2019 in relation to this effect. Both studies found that topical administration of *Aloe vera* as gel or cream did not reduce the prevalence and severity of radiotherapy-induced dermatitis and skin toxicity in breast cancer patients compared to control group [57,58].

## 4. Anti-Inflammatory Activity

Most recent studies on anti-inflammatory activity of *Aloe vera* are focused on the action mechanism of isolated compounds in murine macrophage RAW264.7 cells and mice stimulated with LPS. Hence, the potential anti-inflammatory effect of aloin is related to its ability to inhibit cytokines, ROS production, and JAK1-STAT1/3 signaling pathway [59,60]. Moreover, aloe-emodin sulfates/glucuronides (0.5 μM), rhein sulfates/glucuronides (1.0 μM), aloe-emodin (0.1 μM), and rhein (0.3 μM) inhibited pro-inflammatory cytokines and nitric oxide production, iNOS expression, and MAPKs phosphorylation [61].

In another study, Thunyakitpisal et al. [62] demonstrated that acemannan increased IL-6 and IL-8 expression and NF-κB/DNA binding in human gingival fibroblast via a toll-like receptor signaling pathway. Since there is a relation between high IL-1β levels and periodontal diseases, Na et al. [63] investigated the anti-inflammatory properties of aloin in human oral KB epithelial cells stimulated with saliva from healthy volunteers. This study revealed that those saliva samples with high content in IL-1β stimulated IL-8 production in KB cells, and pretreatments with aloin inhibited IL-8 production by decreasing p38 and extracellular signal-regulated kinases pathway.

In addition to isolated compounds, Ahluwalia et al. [64] evaluated the activity of AVH200^®^, a standardized *Aloe vera* extract which contains alin and acemannan on the activation, proliferation, and cytokine secretion of human blood T cells obtained from healthy individuals aged 18–60, and they found that it decreased CD25 and CD3 expression on CD3(+) T cells. Moreover, AVH200^®^ exhibited concentration-dependent T cell proliferation suppression and IL-2, IFN-γ, and IL-17A reduction. Moreover, the anti-inflammatory effect of *Aloe vera* has also been investigated in an acetaminophen-induced hepatitis (inflammatory condition of the liver) mice model. The results of this study revealed that *Aloe vera* (150 mg/kg) reduced hepatic MDA, IL-12, and IL-18 levels and ALT and increased GSH content [65].

## 5. Anticancer Effects

Studies conducted in the years of the review of this work focusing on cancer are mostly in vitro and in vivo studies. In vitro studies have the main purpose of identifying potential molecules with cytotoxic activity for later evaluation in in vivo studies and clinical trials. In addition, in vitro studies allow elucidating the mechanism of action by identifying promising pharmacological targets. In vivo studies allow us to understand the pharmacological activity and behavior in living organisms prior to their study in humans. Since clinical trials are very limited, and as it is not possible to confirm the anti-cancer activity of *Aloe vera* and its bioactive principles, it would be interesting for future research to focus on this activity based on the promising in vitro and in vivo results.

In vitro and in vivo studies included in the present review are aimed at evaluating cytotoxic and antitumor activity against a variety of cancer types using a diversity of cell lines and animal models (breast and gynecological cancers such as cervical cancer and ovarian cancer, malignant conditions of the gastrointestinal tract (i.e., oral cavity, esophagus, colon) and accessory digestive organs (pancreas), osteosarcomas, and melanoma). One clinical trial focused on the efficacy of *Aloe vera* on ocular surface squamous neoplasia; this clinical trial has been included at the end of this section.

MCF-7 cells, which express estrogen receptor, are the most popular breast cancer cell line, and the immortal HeLa cell line are the oldest and most used cervical cancer cells [66,67]. *Aloe vera* crude extracts (40%, 50%, and 60% for 6, 24, and 48 h) reduced cell viability of cancer cell lines (human breast MCF-7 and cervical HeLa) through apoptosis induction (chromatin condensation and fragmentation and apoptotic bodies appearance in sub-G0/G1 phases) and modulation of effector genes expression (an increase in cyclin D1, CYP1A1, and CYP1A2 expression and a decrease in p21 and bax expression) [68]. Moreover, the isolated compound aloe-emodin has resulted to be an effective anticancer agent against both MCF-7 cells and HeLa cells by inducing mitochondrial and endoplasmic reticulum apoptosis and inhibiting metastasis oxidative stress [69,70,71,72]. Furthermore, a recent study demonstrated that *Aloe vera* extract (300 mg/kg) and training (swimming) combined exerted a protective anticancer effect in mice with breast cancer by inhibiting the COX pathway (COX-2 reduction levels) and prostaglandin E2 production [73]. Finally, aloesin reduced tumor growth in in vitro and in vivo models of ovarian cancers by inhibiting the MAPK signaling pathway [74].

For malignant conditions of gastrointestinal tract and accessory digestive organs, emodin (10, 20, 30, 40 μM for 24 and 48 h) decreased cell proliferation and Bcl-2 protein levels and increased caspase-3 protein expression and Bax protein levels in human oral mucosa carcinoma KB cells [75]. Moreover, aloe-emodin has been shown to effectively suppress esophageal TE1 cancer cells in a concentration-dependent manner (from 2.5 µM to 20 µM concentrations assayed) through inhibiting AKT and ERK phosphorylation and reducing the number of cells in the S phase [76]. Furthermore, *Aloe* polysaccharide induced autophagy alone and in combination with radiation in pancreatic carcinoma BxPC-3 cells as evidenced in ULK1 mRNA expression upregulation and BECN1 and BCL-2 mRNA expression downregulation [77]. Finally, several in vitro and in vivo studies were performed to evaluate the potential anticancer properties of *Aloe vera* and its isolated compounds in colon cancer (fourth most common cancer and the third leading death cause) [78]. Chen et al. [79] exhibited cytotoxic properties of aloe-emodin on colon cancer cells at 10, 20, and 40 μM concentrations through activating the apoptotic pathway, increasing ROS production, and cytosolic calcium levels and up-regulating ER stress-related proteins. Moreover, *Aloe vera* powder and extract 1% and 3% protected C57BL/6J mice from aberrant crypt foci colorectal cancer by increasing hepatic phase II enzyme glutathione S-transferase mRNA levels [80]. Furthermore, *Aloe vera* gel (200 or 400 mg/kg/day orally) reduced inducible NO synthase and COX2 expression, NF-kB activation, and cell cycle progression, inducing cellular factors in BALB/c female mice with induced colitis-associated colon carcinogenesis.

Osteosarcomas are uncommon bone tumors in which malignant cells produce osteoid [81]. Aloe-emodin has also resulted to be a promising photosensitive agent against the human osteosarcoma MG-63 cell line via ROS/JNK signaling pathway as evidenced in an increase of caspases, cytochrome c, CHOP, and GRP78 expression [82,83].

For melanoma (malignant transformation of melanocytes), aloe-emodin protected against metastatic human melanoma cells by decreasing cell proliferation, increasing cell differentiation, and transamidating activity of transglutaminase and dabrafenib antiproliferative activity [84,85].

Regarding clinical trials conducted in recent years on anticancer activity, Damani et al. [86] reported the efficacy of *Aloe vera* eye drops 3 times daily for 3 months in the regression of ocular surface squamous neoplasia in a 64-year-old Hispanic woman. On the other hand, Koo et al. [87] stated that aloe polysaccharide could reduce tobacco associated diseases such as cancer due to its ability to increase urinary excretion of benzo(a)pyrene and cotinine.

## 6. Antidiabetic Effect

Diabetes is a chronic disease presenting with high levels of glucose in blood because of an insulin resistance or an insulin deficiency. Studies on the effect of *Aloe vera* in diabetes and related complications have been investigated mainly in animal models induced by streptozotocin. Consistent evidence supports that oxidative stress is a main cause of the beginning and the progression of diabetes complications such as nephropathies and neuropathies. Hence, using this experimental model, *Aloe vera* showed to reduce blood glucose levels, to increase insulin levels, and to improve pancreatic islets (number, volume, area, and diameter) [88], and this medicinal plant protected from oxidative stress-induced diabetic nephropathy and anxiety/depression-like behaviors [89]. Moreover, *Aloe vera* topical administration (60 mg/mL, four times daily for 3 days of eye drops) favored corneal re-epithelialization in streptozotocin-induced diabetic Wistar rats with corneal alkali burn injury [90]. Furthermore, experiments with genetically modified animals have revealed that *Aloe vera* polysaccharides (100 µg/g for 3 weeks) are responsible for the decrease of blood glucose levels [91]. A recent in vitro study showed that the action mechanism of *Aloe vera* polysaccharides antidiabetic effect is related to its ability to inhibit apoptosis and endoplasmic reticulum stress signaling [91]. In another in vitro study using a high-glucose-induced toxicity cell model, the compound aloe-emodin (20 μM) protected RIN-5F cells derived from rat pancreatic β-cells from glucotoxicity through an apoptotic and anti-inflammatory effects [92]. Lastly, the intake of *Aloe vera* (300 mg twice day for 4 weeks) decreased fasting blood glucose in pre-diabetic subjects [93].

## 7. Antioxidant Properties

Antioxidants are compounds that prevent or slow down biomolecule oxidative damage caused by ROS through free radical scavenging, metal chelation, and enzyme regulation [94]. Kumar et al. 2017 [95] investigated the potential antioxidant activity of crude methanolic extracts of *Aloe vera* from six agro-climatic zones of India using different in vitro methods (i.e., DPPH, metal chelating, and reducing power assay). Antioxidant activity was higher in those species collected in Northern India than in Southern India, which is related to a high content in alkaloids, glycosides, phenolic compounds, flavonoids, and saponin glycosides. Moreover, *Aloe vera* ethanol extract protected, particularly human microvascular endothelial cells, against hydrogen peroxide and 4-hydroxynonenal-induced toxicity by reducing ROS production and HNE-protein adducts formation [96]. The antioxidant activity of *Aloe vera* is, at least in part, due to anthraquinones and related compounds (10 µM) which possess peroxyl radical scavenging activity and reducing capacity [97].

Apart from these in vitro assays, in a clinical trial with 53 healthy volunteers, the intake of *Aloe vera* gel extract (14 days) increased total antioxidant capacity of plasma of subjects [98].

## 8. Bone Protection

In vitro studies with isolated *Aloe vera* compounds have been aimed at studying the potential protective effect on bone pathogenesis. Aloe-emodin induced chondrogenic differentiation on clonal mouse chondrogenic ATDC5 cells which is related to bone formation through BMP-2 and MAPK-signaling pathway activation [99]. Moreover, aloin has resulted to be beneficial in osteoporosis and osteopenia disorders by suppressing receptor activator of NFĸB ligand (RankL) induced through NF-κB inhibition in mouse macrophage RAW 264.7 cells [100,101].

## 9. Cardioprotective Effect

In vivo models of ischemia-reperfusion injury are commonly employed to evaluate the cardioprotective activity of *Aloe vera*. *Aloe vera* administered with gastric gavage previous to abdominal aorta and spinal cord ischemia increased antioxidant enzymes activity (SOD, CAT, and GPx) and reduced lipid peroxidation level (MDA content), edema, hemorrhage, and inflammatory cell migration in Wistar albino rats [102,103]. Moreover, barbaloin, also known as aloin, (20 mg/kg/day, 5 days) administered intragastrically reduced myocardial oxidative stress and inflammatory response and increased AMPK signaling in Sprague-Dawley rats in a myocardial ischemia/reperfusion injury [104]. Esmat et al. [105] demonstrated that this compound (50 mg/kg body weight, twice weekly over 2 weeks), administered intramuscularly, had non-atherogenic activity and iron chelating properties. Another compound isolated from *Aloe vera* and investigated for its cardioprotective properties is aloe-emodin. In an in vitro model of heme protein (hemoglobin), it was demonstrated that aloe-emodin (100 μM) had its maximum activity as an anti-aggregatory agent as evidenced in structural alterations of β sheet and the appearance of α helices [106]. On the other hand, an in vivo study revealed that aloe-emodin could alleviate hyperlipidemia by reducing total cholesterol and low-density lipoprotein-cholesterol levels at doses of 50 and 100 mg/kg for 6 weeks in male Wistar rats [107]. Regarding clinical studies, a double-blind randomized controlled trial showed that *Aloe vera* 300 mg and 500 mg/twice day for 4 and 8 weeks reduced HbA1C, total cholesterol, LDL, and triglyceride levels in pre-diabetic patients [92]. Furthermore, the oral gavage administration of *Aloe vera* (30 mg/kg/day for 1 month) resulted to decrease ischemic fiber degeneration by preventing the formation of lipid peroxides, increasing antioxidant enzymes, and up-regulating the transcription factor NRF1 in Wistar albino rats [108].

## 10. Antimicrobial and Prebiotic Activity

Different studies have been carried out to evaluate the antimicrobial activity of *Aloe vera* and its main constituents. Most of these studies are in vitro and focus on the antibacterial activity. One of the most studied bacteria are *Staphlococcus aureus* and *Pseudomonas aeruginosa*. Hence, *Aloe vera* aqueous extract reduced growth and biofilm formation against methicillin resistant *Staphylococcus aureus* [109]. Moreover, this bacteria has also been inhibited by *Aloe vera* gel (50% and 100% concentrations), along with other oral pathogens obtained from patients with periapical and periodontal abscess including *Actinobacillus actinomycetemcomitans*, *Clostridium bacilli*, and *Streptococcus mutans* using disc diffusion, micro-dilution, and agar dilution methods [110]. One of the compounds attributed to antibacterial activity against *Staphylococcus aureus* is aloe-emodin which acts by inhibiting biofilm development and extracellular protein production [111]. In the case of *Pseudomonas aeruginosa*, *Aloe vera* extracts have shown to inhibit the growth of multidrug-resistant *Pseudomonas aeruginosa* isolated from burned patients with wounds infections at MIC_50_ and MIC_90_ values of 200 µg/mL [112]. *Pseudomonas aeruginosa* growth and biofilm formation inhibition has been also demonstrated for *Aloe vera* inner gel. This *Aloe vera* inner gel also inhibited other Gram-negative bacteria (*Helicobacter pylori* and *Escherichia coli*) as well as the fungus *Candida albicans* [113]. Moreover, in another study, *Aloe vera* hydroalcoholic extract showed antibacterial activity against *Enterococcus faecalis*, an infecting microorganism of the root canals of teeth, with inhibition zones of 13 mm (saturated) and 9.6 mm (diluted) [114]. Furthermore, concentrations up to 1 mg/mL of *Aloe vera* aqueous extracts could inhibit *Mycobacterium tuberculosis* growth, which is the pathogen responsible for causing tuberculosis, one of the most lethal infectious diseases worldwide [115]. Finally, in a clinical trial with 53 healthy volunteers, the daily drinking of *Aloe vera* gel extract for 14 days exerted an antimicrobial activity as shown in a reduction of *Lactobacillus* spp. number [98].

Antiviral activity of *Aloe vera* has been investigated for herpes simplex virus type 1 and H1N1 subtype influenza virus. *Aloe vera* extract gel (concentrations from 0.2% to 5%) showed antiviral activity against herpes simplex virus type 1 on Vero cells by inhibiting its growth [116]. On the other hand, in vitro studies have demonstrated that *Aloe* polysaccharides decreased H1N1 subtype influenza virus replication and viral adsorption period by interacting with influenza virus particles. Moreover, in vivo studies with SPF BALB/c mice infected with PR8(H1N1) improved clinical symptoms and lung damage [117].

The parasite *Plasmodium falciparum* is the main causative agent of malaria, in its most aggressive and lethal form. Kumar et al. [118] investigated the activity of *Aloe vera* crude aqueous extracts collected in six different climatic regions of India (highland, semi-arid, arid, humid subtropical, tropical wet and dry, and humid subtropical climate) against a chloroquine-sensitive strain of *Plasmodium falciparum*. This study showed that those *Aloe vera* from colder climatic regions possessed the highest antiplasmodial activity which was related to the highest aloin and aloe-emodin content (EC50 value of 0.289 µg/mL).

Finally, there are other studies which support the prebiotic potential of *Aloe vera* defined as “a substrate that is selectively utilized by host microorganisms conferring a health benefit”. *Aloe vera* mucilage (rich in acemannan) could improve gastrointestinal health by increasing short chain fatty acids and modifying bacterial composition [119]. Moreover, acemannan and fructans from *Aloe vera* increased bacterial growth, especially *Bifidobacterium* spp. population [120].

## 11. Other Effects

*Aloe vera* has also been investigated for treating reproductive health care problems. The results of these works carried out with experimental animals are contradictory. While Asgharzade et al. [121] demonstrated that *Aloe vera* ethanol extract (150 and 300 mg/kg) had negative effects on spermatogenesis and sperm quality in Wistar rats, Erhabor and Idu [122] observed that *Aloe vera* ethanol extract (400 mg/kg) improved male sexual behavior (mount frequency and latency, intromission frequency and latency, and testosterone levels) and Behmanesh et al. [123] that *Aloe vera* extract increased body and testis weights, spermatocyte and spermatids quantity, and seminiferous tubule diameter and height.

*Aloe vera* processed gel prevented of ovoalbumin-induced food allergy by exerting an anti-inflammatory action (histamine, mast cell protease-1, and IgE reduction) [124].

At the blood level, the oral administration of *Aloe vera* gel prevented and restored lymphopenia and erythropenia as well as IgA secretion on cyclophosphamide-induced genetically modified mice [125]. Moreover, *Aloe vera* ethanol extract (200 mg/kg, 400 mg/kg, and 600 mg/kg) normalized levels of white blood cells, red blood cells, and platelet count through antioxidant mechanisms [126].

Regarding diseases of the musculoskeletal system, aloe-emodin showed to reduce viable cell numbers (concentrations ≥10 µM) and to induce apoptosis by arresting G2/M phase (concentrations ≥20 µM) in MH7A human synovial fibroblast-like cells, aloe-emodin being a promising agent to treat rheumatoid arthritis and a complementary treatment to methotrexate [127]. Moreover, *Aloe vera* lyophilized extract ointment reduced tendon lesions and increased non-collagenous proteins in Wistar rats with partial transection of the calcaneal tendon [128].

The dose of 10 mg/kg of *Aloe vera* aqueous extract (3 times daily for a week) resulted to be the most effective in morphine withdrawal syndrome in morphine-dependent female rats as shown in agitation, disparity, and floppy eyelids reduction [129].

Finally, highlighting the protective effect of *Aloe vera* gel extract (seven weeks, 500 mg/kg b.w. daily) on pulmonary tissue of cigarette smoke induced in Balb/c mice by reducing mucin production, citrulline and NO levels, and peroxidative damage [130].

## 12. Conclusions

*Aloe vera* has been traditionally used to treat skin injuries (burns, cuts, insect bites, and eczemas) and digestive problems because of its anti-inflammatory, antimicrobial, and wound healing properties. Research on this medicinal plant has been aimed at validating traditional uses and deepening the mechanism of action, identifying the compounds responsible for these activities. Likewise, new actions have been investigated for *Aloe vera* and its active compounds, especially highlighting its promising role as a cytotoxic, antitumoral, anticancer, and antidiabetic agent. In the last 6 years, most pharmacological studies have been in vitro and in vivo works. Among in vitro studies, antimicrobial, anti-inflammatory, cytotoxic, antitumor, anticancer, and skin protection activities are the most studied in number. It should be especially noted that among in vitro studies there are several works that evaluate the protective action of *Aloe vera* in bone diseases such as osteoporosis. The results on bone protection are promising; however, it is necessary to perform them with experimental animals and humans. Regarding in vivo studies, these are aimed at evaluating cardioprotective effect, cytotoxic, antitumor and anticancer activities, and skin protection activities. Compared to in vitro and in vivo assays, clinical trials are limited and focus on digestive and skin protective effects. In addition, these clinical trials have been conducted just with *Aloe vera*, but not with its isolated compounds; therefore, it would be of interest to study the clinical effect of relevant metabolites in different human conditions and pathologies. Among the major active compounds, research in the last six years focused on aloe-emodin, aloin, aloesin, amodin, and acemannan. Of these, aloe-emodin and aloin have been the most studied ones. Particularly, aloe–emodin has resulted to be a promising agent as an antimicrobial, antidiabetic, cytotoxic, cardiprotective, and bone protective (in in vitro studies) as well as anti-inflammatory and skin protective compound (in in vivo studies). Aloin was effective in inflammatory process and bone diseases (in vitro studies) and in cancer and cardiovascular diseases (in vivo studies). The promising results of basic research encourage a greater number of clinical trials to test the clinical application of *Aloe vera* and its main compounds, particularly on bone protection, cancer, and diabetes.

**Table 1:** In vitro pharmacological studies for *Aloe vera*.

*Aloe Vera* Composition	Experimental Model	Major Findings	References
Digestive Diseases Protection			
Acemannan	Human deciduous pulp cells	↑ Pulp cell proliferation ↑ ALP ↑ Type I collagen ↑ BMP-2, BMP-4, vascular endothelial growth factor and dentin sialoprotein expression	[6]
*Aloe* polysaccharide	HT-29 cells LPS and TNF-α induced	↑ JAK2 and STAT-3 expression ↓ JAK2, p-JAK2, STAT-3 and p-STAT3 protein expression Ulcerative colitis protection	[5]
*Aloe vera* extract	Dental pulp stem cells from rabbits	↑ Cell viability	[4]
Skin Protection			
*Aloe* polysaccharide	HaCaT cells	↓ TNF-α levels ↓ IL-8 and IL-12 expression levels ↓ p65 expression ↑ *IkB*-alpha protein expression Psoriasis protection	[49]
*Aloe vera*	HEKa and NFDH cells	↑ Cell viability ↑ Cell proliferation ↑ Cell migration ↑ Wound healing	[31]
*Aloe vera*	HaCaT cells	↓ Photodamage Membrane integrity maintenance ↑ Lysosomal stability	[32]
*Aloe vera* ethanolic extract	c147 cells	↑ Fibroblast migration ↑ VEGF-A gene expression ↑ Wound healing	[30]
*Aloe vera* gel	Mouse embryonic fibroblast cells	↑ TFGβ1 and bFGF factor expression ↑ Wound healing	[29]
*Aloe vera* gel	HEKa Human skin equivalent model	↑ Cell number ↑ Wound healing ↑ Keratinocyte proliferation and differentiation ↑ Cell surface expression of adhesion molecules (β1-integrin, α6-integrin, β4-integrin and E-cadhesin) ↑ Wound healing	[28]
Aloesin	HaCaT cells	↑ Cell migration ↑ Cytokines and growth factors ↑ Wound healing	[35]
Aloin	Hs 68 cells Heat stress-mediated oxidative stress	↑ GSH ↑ SOD activity ↓ Lipid peroxidation ↓ 8-OH-dG ↑ Cell viability ↓ ROS	[34]
Aloin	κβ cells	↓ IL-8 production	[63]
Emodin	THP-1 cells and HaCaT cells	↑ VEGF ↑ MCP-1 Burn wound protection	[5]
Pure *Aloe vera* gel	Primary cultures of corneal epithelial cells and fibroblasts	↑ Corneal epithelial cell wound closure (*Aloe vera* concentrations ≤ 175 μg/mL) ↑ Type IV collagen-degrading activity	[33]
Anti-Inflammatory Activity			
Acemannan	Human gingival fibroblasts	↑ IL-6 and IL-8 expression ↑ NF-Κβ/DNA binding	[62]
*Aloe vera* extract (AVH200® Batch: 2013016)	Peripheral blood mononuclear cells	↓ CD25 and CD28 expression Suppression of T cell proliferation ↓ IL-2, IFN-γ and IL-17A secretion	[61]
Aloe-emodin sulfates/glucuronides, rhein sulfates/glucuronides, aloe-emodin and rhein	RAW 264.7 cells stimulated with LPS and mouse peritoneal excluded macrophages	↓ iNOS expression ↓ TNF-α, IL-12, and NO production ↓ MAPKs phosphorylation	[64]
Aloin	RAW 264.7 cells	↓ iNOS expression ↓ IL-1β, IL-6, tumor necrosis factor alpha and NO dose-dependently ↓ JAK1-STAT1/3 activation ↓ STAT1/3 nuclear translocation ↓ ROS production	[59]
Aloin	KB cells	↓Salivary IL-1β-induced IL-8 production ↓p38 and ERK pathway	[63]
Barbaloin/aloin	RAW 264.7 cells stimulated with LPS	↓ Phosphorylation levels of IκBα and NF-κB p65 ↓ Pro-inflammatory cytokines (TNF-α, IL-1β and IL-6) expression ↓ ROS	[60]
Anticancer Effects			
Aloe polysaccharide	BxPC-3 cells	↑ ULK1 mRNA expression ↓ BECN1 and BCL-2 mRNA expression	[77]
*Aloe vera* crude extract	MCF-7 cells and HeLa cells	↓ Cell viability Apoptosis induction ↓ Cyclin D1, CYP1A1 and CYP1A2 ↑ Bax and p21 expression	[68]
Aloe-emodin	Metastatic human melanoma cell lines Primary stem-like cells	↓ Cell proliferation ↑ Cell differentiation ↑ Transamidating activity of transglutaminase ↑ Dabrafenib antiproliferative activity	[85]
Aloe-emodin	TE1 cancer cells	↓ AKT and ERK phosphorylation ↓ Number cells in S phase	[76]
Aloe-emodin	MCF-7 cells Photodynamic therapy	↓ Adhesion, migration and invasion of cells cytoskeleton disorganization Apoptosis: mitochondrial and endoplasmic reticulum death pathways	[70]
Aloe-emodin	HUVECs cells Photodynamic therapy	↓ Angiogenesis and Cell Metastasis MAPK Signaling Pathway activation ↓ Adhesion, migration and invasion of cells Apoptosis: mitochondrial death pathways cytoskeleton disorganization	[79]
Aloe-emodin	SW620 and HT29 cells	↓ Cell viability ↑ Apoptosis (Upregulation of CHOP and caspase 12) ↑ ROS Upregulation of unfolded protein response proteins	[79]
Aloe-emodin	HeLa cells	↓ Cell proliferation G2/M and S phase cell cycle arrest ↑ Radiosensitivity ↑ Cyclin B and γ-H2AX expression ↑ ALP activity	[69]
Aloe-emodin	MG-63 cells	↑ ROS production ↓ Mitochondrial membrane potential ↑ Caspase-3, caspase-9, caspase-12 expression ↑ Cytochrome c release	[82]
Aloe-emodin	HeLa cells	↑ Mitotic death ↓ Mitotic index ↓ G2/M phase	[72]
Aloe-emodin	Breast cancer cells (MCF-7, MDA-MB-231, MDA-MB-468, BT-474, HCC-1954)	↑ Tamoxifen cytotoxicity	[71]
Aloe-emodin	MG-63 cells	↓ Cell viability ↑ Autophagy ↑ Apoptosis ↑ ROS	[83]
Aloesin	SKOV3 cells	↓ Cell viability ↓ Cell clonality ↓ Cell cycle at S-phase ↑ Apoptosis ↓ Migration and invasion cancer	[74]
Emodin	KB cells	↓ Cell proliferation ↑ Caspase-3 upregulation ↑ Bax protein levels ↓ Bcl-2 protein levels	[75]
Antidiabetic Effect			
*Aloe vera* polysaccharides	Hamster pancreatic β-cell line HIT-T15 in response to free fatty acids	↓ Number of apoptotic β-cell death Relief of endoplasmic reticulum stress signaling	[91]
Aloe-emodin	RIN-5F cells High glucose induced toxicity	↑ Cell viability ↓ ROS generation ↓ Pro-inflammatory cytokines levels (IFN-γ, IL-1β) ↑ Anti-inflammatory cytokine levels (IL-6 and IL-10) ↓ DNA fragmentation ↓ Bax, caspase 3, Fadd, and Fas expression ↑ Bcl-2 expression	[92]
Antioxidant Properties			
*Aloe vera* crude methanolic extracts	In vitro antioxidant methods: DPPH, metal chelating, hydrogen peroxide scavenging, reducing power and β-carotene-linoleic	Antioxidant activity	[95]
*Aloe vera* ethanol extracts	Cell models (HeLa, HMEC, HaCat, and HOS) hydrogen peroxide and 4-hydroxynonenal induced	↓ ROS production ↓ HNE protein adducts HMEC cells were the most sensitive	[96]
Anthraquinone derivatives Phenolic derivatives Chromones Pyrones	Peroxyl radical scavenging Reducing capacity	Antioxidant activity	[97]
Bone Protection			
Aloe-emodin	ATDC5 cells	↑ Accumulation cartilage nodules ↑ Matrix proteoglycans synthesis ↑ ALP activity ↑ Chondrogenic marker genes ↑ ERKs ↑ BMP-2 protein expression	[99]
Aloin	RAW 264.7 cells	↓ RankL induced miR-21 expression ↓ Cathepsin K Osteoporosis protection	[101]
Aloin	RAW264.7 cells	↓ TRAP content ↑ F4/80 content ↓ Cathepsin K ↓ RANKL-induced NF-κB pathway ↓ DNA binding activity of NF-κB Osteoporosis protection	[100]
Cardioprotective EFFECT			
Aloe emodin	Model heme protein (hemoglobin)	↓ Hemoglobin aggregation (máximum effect at 100 μM)	[106]
Antimicrobial and Prebiotic Activity			
Acemannan and fructans	*Lactobacillus* and *Bifidobacterium* species Human fecal bacteria	↑ Bacterial growth (fructans) ↑ *Bifidobacterium* spp population ↑ Acetate concentrations	[120]
*Aloe* polysaccharides	H1N1 subtype Influenza A virus	↓ H1N1 subtype influenza virus replication ↓ Viral adsorption period	[117]
*Aloe vera* aqueous extract	Methicillin resistant *Staphylococcus aureus*	↓ Growth ↓ Biofilm formation	[109]
*Aloe vera* aqueous extracts	Drug resistant *Mycobacterium tuberculosis*	Inhibition zone: 60 mm (disk diffusion method) ↓ Cell growth (up to 1 mg/mL concentration) (pour plate method)	[115]
*Aloe vera* crude aqueous extracts	*Plasmodium falciparum*	Antiplasmodial activity	[95]
*Aloe vera* extracts	Multidrug-resistant *Pseudomonas aeruginosa*	Similar MIC_50_ and MIC_90_	[112]
*Aloe vera* gel	*Actinobacillus actinomycetemcomitans Clostridium bacilli*, *Streptcoccus mutans* and *Staphlococcus aureus*	Antibacterial activity against oral pathogens	[110]
*Aloe vera* gel	Virus herpes simplex 1	↓ HSV-1 growth	[116]
*Aloe vera* hydroalcoholic extract	*Enterococcus facecalis*	Antibacterial activity against *Enterococcus faecalis*	[114]
*Aloe vera* inner gel	Gram negative bacteria, Gram positive bacteria and *Candida albicans*	Antimicrobial and antibiofilm activities against Gram negative bacteria (*Helicobacter pylori*, *Escherichia coli* and *Pseudomonas aeruginosa*) and *Candida albicans*	[113]
*Aloe vera* mucilage	Intestinal microbiota from healthy donors	Prebiotic activity (↑ short chain fatty acids and modifications in bacterial composition)	[119]
Aloe-emodin	*Staphylococcus aureus*	↓ Biofilm development (initial adhesion and proliferation stages) and extracellular protein production	[111]
Other Effects			
Aloe-emodin	MH7A human RA synovial fibroblast-like cells	↓ Viable cells number ↑ Apoptosis (G2/M phase arrest) Rheumatoid arthritis protection	[127]
Aloe-emodin	ARPE-19 cells	↓ VEGF secretion ↓ VEGFA and PHD-2 mRNA expression ↓ VEGFA, HIF-1α and PHD-2 protein expression	[131]

**Table 2:** In vivo pharmacological studies for *Aloe vera*.

*Aloe Vera* Composition	Experimental Model	Major Findings	References
Digestive Diseases Protection			
Acemannan	Beagle Dogs	Mineralized Bridge Formation	[6]
*Aloe* polysaccharide	2,4,6-three nitrobenzene sulfonic acid colitis induced Rats	↑ JAK2, p-JAK2, STAT-3 and p-STAT3 protein expression ↓ Ulcerative colitis	[5]
*Aloe vera* gel	Balb/c mouse model of alcohol-induced acute gastritis	↑ Matrix metalloproteinase-9 inhibitory activity	[24]
*Aloe vera*	Male Wistar rats Irradiated model	↑ Salivary flow rate	[7]
Skin Protection			
Aloe emodin	BALB/c mice burn wound-induced	↑ Wound healing activity (↑ re-epithelialization and angiogenesis)	[6]
Aloe sterols	Female HR-1 hairless mice Ultraviolet B-induced skin photoaging	↓ Skin dryness, epidermal thickness and wrinkle formation ↓ Dermal collagen fibers degeneration ↓ Cutaneous apoptosis cells ↓ Pro-inflammatory cytokines ↓ Matrix metalloproteinases	[43]
*Aloe vera*	Adult male Wistar rats with incision on neck	↑ Fibroblasts ↑ TGF-β gene expression	[38]
*Aloe vera*	Adult female Sprague Dawley rats with a skin wound infected with methicillin-resistant *Staphylococcus aureus*	↓ Inflammatory cell infiltration ↑ Wound closure and skin tensile strength % ↑ Collagen deposition ↑ Skin tensile strength	[132]
*Aloe vera* aqueous gel extract	X-ray irradiated Male balb/c mice	↑ Hepatic and renal function parameters ↓ROS ↓ Lipid peroxidation ↑ GSH ↑ GR, GPx, CAT, SOD, GST ↑ Sperm count/motility and testosterone levels	[45]
*Aloe vera* cream	Male Sprague-Dawley rats	↓ Wound percentage ↓ Leucocytes infiltration ↓ Angiogenesis ↓ CD8^+^ lymphocytes expression ↑ Epidermal thickness ↑ CD4^+^ lymphocytes expression	[39]
*Aloe vera* extract gel	Wistar rats with a wound made by incision	↑ Organization of skin and collagen	[36]
*Aloe vera* gel	Male Wistar rats Alloxan-induced diabetes with wounds	↓ Necrotic tissue and inflammation ↓ Wound areas Better reepithelialization	[40]
*Aloe vera* gel	Male Wistar rats	↑ Wound contraction and epithelialization ↓ Scar tissue size ↑ Alignment and organization of regenerated scar tissue	[37]
*Aloe vera* gel aqueous extract	X-ray irradiated Male balb/c mice	↑ Hepatic and renal function parameters ↓ ROS, ↓ Lipid peroxidation ↓Lactate dehydrogenase	[45]
*Aloe vera* gel powder	Ovariectomy HR-1 hairless mice UV-irradiation model	↓ Matrix metalloproteinases (MMPs) expression ↑ Epidermal growth factor ↑ Hyaluronan synthase	[41]
*Aloe vera* gel powder	HR-1 hairless mice UVB-induced model	↑ Skin elasticity ↓ Matrix metalloproteinase 2, 9 and 13 ↑ Hyaluronic content ↑ HA synthase 2	[42]
*Aloe vera* hydroalcoholic extract	Wistar rats with traumatic ulcers	No acceleration of oral wound	[133]
Aloesin	SKH-1 hairless mice	↑ Angiogenesis ↑ Collagen deposition and granulation tissue formation	[35]
Anti-Inflammatory Activity			
*Aloe vera*	Male ICR strain mice Acetaminophen-induced hepatitis	↓ Hepatic MDA ↓ IL-12 and IL-18 ↓ ALT transaminase ↓ Hepatitis	[65]
Aloe-emodin sulfates/glucuronides, rhein sulfates/glucuronides, aloe-emodin and rhein	LPS-induced septic mice	↓ NO level	[61]
Barbaloin	BALB/c mice LPS-induced acute lung injury	Histological analysis revealed certain protective effect	[60]
Anticancer Effects			
*Aloe vera* aqueous extract	Male Swiss albino mice	Mutagenic activity Cytotoxic effect Wound healing (antioxidant properties)	[47]
*Aloe vera* extract	Mice with breast cancer by implantind	↓ COX-2 level ↓ VEGF levels	[73]
*Aloe vera* powder and extract	C57BL/6J mice high-fat diet induced and azoxymethane induced aberrant crypt foci colorectal cancer	↑ Hepatic phase II enzyme glutathione S-transferase mRNA levels ↓ Cell proliferation in the colonic mucosa ↓ Number of aberrant crypt foci	[80]
*Aloe vera* gel	BALB/c female mice with induced colitis-associated colon carcinogenesis	↓ Multiplicity of colonic adenomas and adenocarcinomas ↓ Adenoma and adenocarcinoma development ↓ Activation of nuclear factor kappa B ↓ Inducible nitric oxide synthase and cyclooxygenase-2 expression ↓ Cell cycle progression-inducing cellular factors	[125]
Aloesin	Mice	↓ Tumor growth	[74]
Aloin	Male Swiss albino rats	↑ Erythropoiesis impairment ↑ Serum iron level No changes on serum lipid profile No changes on serum elements and kidney function parameters	[105]
Emodin	SPF BALB/c-nu nude mice	↑ Survival time of tumor ↓ Effect on transplantation tumors ↓ Oral cancer	[75]
Antidiabetic Effect			
*Aloe vera* extract	Streptozotocin-induced diabetic Wistar rats	↓ Blood glucose levels ↑ Insulin levels ↑ Number, diameter, volume and area of pancreatic islets	[88]
*Aloe vera* extract	Streptozotocin-induced nephropathy diabetic Wistar rats	↓ Development of nephropathy ↓ Lipid alteration ↓ Renal oxidative stress Direct renoprotective action	[89]
*Aloe vera* gel	Streptozotocin-induced diabetic male Wistar rats	↓ Anxiety/depression-like behaviors ↑ Exploratory and locomotor activities ↑ Memory performance ↓ Stress related behaviors ↓ Oxidative stress ↑ Neuronal loss in hippocampus	[134]
*Aloe vera* lyophilized extract	Wistar rats with corneal alkali-burn injury Normal rats and diabetic rats streptozotocin-induced	↑ Wound healing (especially in diabetic rats) ↓ Edema Complete and higher corneal re-epithelialization	[90]
*Aloe vera* polysaccharides	C57BL/KsJ-db/db male mice fed with high fat diet	↓ Fasting blood glucose levels	[91]
Cardioprotective Effect			
*Aloe vera*	Wistar albino rats Spinal cord ischemia reperfusion injury model	↓ MDA levels ↓ NF-κβ and nNOS expressions ↓ Hemorrhage ↓ Edema ↓ Inflammatory cell migration ↓ Neurons	[102]
*Aloe vera* extract	Wistar Albino rats with ischemia—reperfusion injury of sciatic nerve	↓ Ischemic fiber degeneration ↓ MDA ↑ NRF1 level ↑ SOD activity	[108]
*Aloe vera* gel	Male Wistar albino rats Ischemia reperfusion injury model	↑ SOD, CAT and GPx ↓ MDA levels	[103]
*Aloe vera* gel	Male Wistar Albino rats Sciatic nerve ischemia model	↓ Ischemic fiber degeneration ↓ MDA content ↑ NRF1 level and SOD activity Neuroprotection	[108]
Aloe-emodin	Male Wistar rats	↓Total cholesterol ↓ Low-density lipoprotein-cholesterol ↓ Hyperlipidemia	[107]
Aloe-emodin	Sprague Dawley rats Hypoxia conditions	↓Hypoxia-induced retinal neovascularization	[131]
Aloin	Swiss albino rats	↓ Triacylglycerols ↓ Total cholesterol ↓ Cholesteryl esters ↓ Low density lipoprotein–cholesterol ↓ Very low density lipoprotein–cholesterol ↓ Urea ↓ Creatinine ↓ Blood hemoglobin concentration ↑ Serum iron level	[105]
Barbaloin/aloin	Sprague-Dawley rats Myocardial ischemia/reperfusion injury model	↓ I/R induced myocardial oxidative stress and inflammatory response ↑ AMPK signaling	[104]
Antimicrobial and Prebiotic Activity			
*Aloe* polysaccharides	PR8(H1N1) virus infection SPF BALB/c mice	Clinical symptoms improvement Lung damage improvement	[117]
Other Effects			
*Aloe vera* aqueous extract	Female Wistar albino rats Morphine dependent model	↓ Agitation, disparity and floppy eyelids	[129]
*Aloe vera* aqueous extract	Wistar rats drug-induced sleeping and anesthesia and analgesia	↑ Loss of righting reflex ↓ Locomotion activity Changes in total sleep time, percent of REM sleep and percent of NREM sleep	[135]
*Aloe vera* ethanol extract	Wistar rats	↑ Mounting frequency ↑ Intromission frequency ↓ Mount and intromission latencies ↑ Ejaculatory latency ↑ Testosterone and cholesterol concentrations	[122]
*Aloe vera* ethanol extract	Rats	↓ TNF-α levels ↓ NK cells ↓ Th17 cells percentage Hepatoprotective	[136]
*Aloe vera* gel	C57BL/6 female mice C3H/HeJ mice	↑ Lymphocyte and erythrocytes number Lymphopenia and erythropenia restoration IgA secretion restoration	[125]
*Aloe vera* gel extract	Wistar rats Bisphenol A Induced Testicular Toxicity	↑ Body and testis weights ↑ Seminiferous tubule diameter and height of seminiferous epithelium ↑ Quantity of spermatocyte and spermatids ↓ MDA ↑ GSH	[123]
*Aloe vera* gel extract	Balb/c mice pulmonary tissue of cigarette smoke induced	↓ Degree of histoarchitectural alterations ↓ Mucin production ↓ NO levels ↓ Citrulline levels ↓ Peroxidative damage ↓ Serum LDH activity	[130]
*Aloe vera* lyophilized extract ointment	Wistar rats with partial transection of the calcaneal tendon	↑ Non-collagenous proteins ↑ Content and arrangement of glycosaminoglycans ↓ Tendon lesions	[128]
*Aloe vera* ethanol	Albino rabbits	Normalized levels of white blood cells, red blood cells, platelet count, packed cell volume, mean cell volume and haemoglobin values ↓ MDA ↑ CAT	[126]
*Aloe vera* gel ethanol extract	Wistar rats	↓ Testes weight ↓ Serum testosterone ↓ Sperm count ↓ Fertility ↑ Serum NO Negative effects on spermatogenesis and sperm quality	[121]
*Aloe vera* processed gel	BALB/c mice on ovalbumin -induced food allergy	↓ Serum concentrations of type 2 helper T cell (Th2) cytokines (Interleukin-(IL)-4, IL-5, and IL-13) ↓ Histamine ↓ Mast cell protease-1 ↓ Immunoglobulin IgE ↑ IL-10 production ↑ Population of type 1 regulatory T (Tr1) cells ↓ Allergy	[124]
Aloin	F344/N Rats	↑ Incidences and severities of mucosal and goblet cell hyperplasia ↑ Shifts in gut microbiota structure	[137]

**Table 3:** Clinical trials with *Aloe vera*.

Reference	Study Design	Number of Patients	Intervention	Results
Digestive Diseases Protection				
Anuradha et al. (2017) [21] India	-	74	Group 1: *Aloe vera* juice (30 mL/twice daily) and *Aloe vera* gel (5 mg/3 times daily) for 3 months Group 2: intralesional injection of hydrocortisone (25 mg/mL) and hyaluronidase (1500 IU) weekly for 6 weeks with antioxidant supplements for 3 months	Oral submucous fibrosis: ↓ Burning sensation ↑ Mouth opening, cheek flexibility, and tongue protrusion
Ashouri Moghaddam et al. (2017) [12] Iran	Single-blind clinical trial	20	Group 1: *Aloe vera* gel Group 2: Placebo (distilled water)	Chronic periodontitis: ↓ Gingival index ↓ Probing depth
Fallahi et al. (2016) [15] Iran	Randomized double blind	20	Group 1: *Aloe vera* Group 2: Placebo	↓ Swelling ↓ Postoperative pain
Gupta et al. (2014) [8] India	Double blind randomized control trial	300	Group 1: *Aloe vera* mouthwash group Group 2: chlorhexidene group Group 3: Placebo	↓ Plaque index and gingival index
Ipshita et al. (2018) [13] India	-	90	Group 1: Placebo Group 2: 1% alendronate gel Group 3: *Aloe vera* gel	Chronic periodontitis: ↑ defect depth reduction
Kalra et al. (2017) [16] India	-	48	Group 1: *Aloe vera* gel Group 2: mineral trioxide aggregate	Pulpotomy: Success rates was higher in mineral trioxide aggregate than in *Aloe vera* gel
Kurian et al. (2018) [14] India	Randomized, single-center, longitudinal, triple-blinded, parallel arm y	90	Group 1: Placebo Group 2: 1% metformin gel Group 3: *Aloe vera* gel 6–12 months	Chronic periodontitis: ↓ Pocket probing depth ↓ Clinical attachment level ↑ Bone fill and regeneration
Mansouri et al. (2016) [20] Iran	Randomized controlled clinical trial	64	Group 1: *Aloe vera* solution (5 mL/two minutes wash, three times daily) Group 2: ordinary mouthwashes. 14 days	Stomatitis: ↓ Chemotherapy-induced Stomatitis in patients with lymphoma and leukemia
Nimma et al. (2017) [17] India	Cross-sectional randomized interventional study	40	Group 1: analgesics (7 days) and socket healing Group 2: *Aloe vera* gel (7 days) and socket healing	Ulcers: ↓ Pain ↑ Healing
Panahi et al. (2015) [23] Iran	Pilot, randomized controlled, open-label, trial	79	Group 1: *Aloe vera* syrup (10 mL/day) Group 2: omeprazole capsule (20 g/day) Group 3: Ranitidine tablet (150 mg twice daily). 4 weeks	↓ Frequency symptoms gastroesophageal reflux disease
Prabhakar et al. (2015) [18] India	Experimental, in vivo intergroup split mouth, randomized clinical trial	10	Group 1: Distilled water Group 2: Propolis extract Group 3: *Aloe vera* extract	Antimicrobial: ↓ Bacterial counts Disinfection
Pradeep et al. (2016) [9] India	Single center, randomized, longitudinal, triple masked, interventional study	60	Group 1: Placebo Group 2: *Aloe vera* gel	Chronic periodontitis: ↓ Plaque index ↓ Modified sulcus bleeding index ↓ Probing depth ↑ Clinical attachment level Patients with type 2 diabetes and chronic periodontitis
Rahmani et al. (2014) [27] Iran	Prospective observational clinical trial	60	Cream of 0.5% *Aloe vera* juice powder (3 times daily) 6 weeks	↓ Chronic anal fissure pain and hemorrhaging upon defection ↑ Wound healing
Sahebjamee et al. (2015) [19] Iran	Triple-blind randomised and controlled interventional	26	Group 1: *Aloe vera* mouthwash Group 2: benzydamine mouthwash 0.15%	Oral mucositis: ↓ Severity of radiation-induced mucositis in patients with head and neck cancers
Sahebnasagh et al. (2017) [25] Iran	Double-blind placebo-controlled trial	20	Group 1: *Aloe vera* gel 3% Group 2: Placebo	Proctitis: Improvement of diarrhea, fecal urgency, clinical presentation total, Radiation Therapy Oncology Group total and lifestyle
Singh et al. (2016) [22] India	-	40	Group 1: *Aloe vera* gel (three times daily) + physiotherapy Group 2: Antioxidant capsules (twice daily) + physiotherapy 3 months	Oral submucous fibrosis: ↓ Burning sensation ↑ Mouth opening, cheek flexibility, and tongue protrusion
Størsrud et al. (2015) [26] Sweden	Randomized, double-blind, placebo controlled study	68	Group 1: *Aloe vera* extract (AVH200®) Group 2: Placebo 4 weeks	Irritable bowel syndrome: ↓ severity of the gastrointestinal symptoms
Vangipuram et al. (2016) [10] India	Randomized controlled trial	390	Group 1: *Aloe vera* mouth wash Group 2: Chlorhexidine (0.12%) mouth wash Group 3: Placebo	Gingivitis: *Aloe vera* has equal effectiveness than chlorhexidine ↓ Plaque index and gingival index
Yeturu et al. (2016) [11] India	Randomized single-center, single-blind, parallel group, controlled trial	85	Group 1: *Aloe vera* mouth wash Group 2: chlorine dioxide mouth wash Group 3: chlorhexidine mouth wash 10 mL of mouth rinse for 1 min, twice daily for 15 days	↓ Mean plaque and gingival scores
Skin Protection				
Ahmadloo et al. (2017) [57] Iran	Prospective randomized controlled clinical trial	100	Group 1: *Aloe vera* gel twice daily Group 2: control	Dermatitis: No positive effect on prevalence or severity of radiation dermatitis
Avijgan et al. (2016) [48] Iran	-	60	Group 1: *Aloe vera* gel twice daily Group 2: Conventional treatment 3 months	Ulcers: wound healing
Burusapat et al. (2018) [53] Thailand	Double-blind, randomized, controlled trial	12	Group 1: *Aloe vera* gel Group 2: Placebo	↑ Split-thickness skin graft donor-site healing No pain relief
Hekmatpou et al. (2018) [50] Iran	Triple-blind randomized clinical trial	80	Group 1: pure *Aloe vera* gel twice daily Group 2: Placebo (gel or water and starch) 10 days	Ulcers: ↓ pressure ulcers
Hoopfer et al. (2015) [58] Canada	Three-Arm Randomized Phase III Trial	248	Group 1: *Aloe vera* cream Group 2: Placebo	No ↓ skin reaction severity in breast cancer radiation therapy
Irani and Varaie (2016) [51] Iran	Randomized clinical trial	30	Burned area: nitrofurazone 2% Symmetry burned area: *Aloe vera* gel	Burns: Earlier epithelialization and granulation tissue
Molazem et al. (2014) [52] Iran	Prospective randomized double-blind clinical trial	90	Group 1: *Aloe vera* gel Group 2: dry gauze alone	↑ Cesarean wound healing
Tanaka et al. (2015) [55] Japan	Randomized, double-blind, placebo-controlled study	58	Group 1: *Aloe* sterol (5 tablets/daily) Group 2: Placebo 12 weeks	↓ Facial wrinkles
Tanaka et al. (2016) [54] Japan	Randomized, double-blind, placebo-controlled study	48	Group 1: *Aloe* sterol (5 tablets/daily) Group 2: Placebo 12 weeks	↑ Skin elasticity in photodamaged skin
Tanaka et al. (2016) [54] Japan	Randomized, double-blind, placebo-controlled study	64	Group 1: *Aloe* sterol-supplemented yogurt Group 2: Placebo	↑ Gross elasticity, net elasticity, biological elasticity, skin fatigue area, collagen content
Anticancer Effects				
Damani et al. (2015) [86] USA	Case report	1	*Aloe vera* eye drops 3 times daily	Ocular surface squamous neoplasia: Lesion regressed
Koo et al. (2019) [87] South Korea	Randomized	40	Group 1: *Aloe* polysaccharide (600 mg/day) Group 2: Propolis (600 mg/day) Group 3: *Aloe* polysaccharide + propolis 4 weeks Group 4: Placebo	Cancer: ↑ Urinary excretion of benzo(a)pyrene and cotinine ↓ Creatinine, glucose, and total bilirubin levels ↓ Risk of cancer associated with tobacco
Antidiabetic Effect				
Alinejad-Mofrad et al. (2015) [93] Iran	Double blind randomized controlled trial Pre-diabetes	72	Group 1: Placebo Group 2: *Aloe vera* 300 mg/twice day (AL300) Group 3: *Aloe vera* 500 mg/twice day (AL500) 4 and 8 weeks	Diabetes: ↓ Fasting blood glucose (AL300, 4 weeks)
Antioxidant Properties				
Prueksrisakul et al. (2015) [98] Thailand	-	53	*Aloe vera* gel extract daily 14 days	Antioxidant: ↑ Plasma total antioxidant capacity
Cardioprotective Effect				
Alinejad-Mofrad et al. (2015) [93] Iran	Double blind randomized controlled trial Pre-diabetes	72	Group 1: Placebo Group 2: *Aloe vera* 300 mg/twice day (AL300) Group 3: *Aloe vera* 500 mg/twice day (AL500) 4 and 8 weeks	↓ HbA1C (AL300, 8 weeks) ↓ Total cholesterol (AL500, 8 weeks) ↓ LDL-C (AL500, 8 weeks) ↓ Triglyceride level (AL500, 4 weeks)
Antimicrobial and Prebiotic Activity				
Prueksrisakul et al. (2015) [98] Thailand	-	53	*Aloe vera* gel extract daily 14 days	Antimicrobial: ↓ *Lactobacillus* spp.
